# Are current clinical guidelines on the use of Peripheral Intravenous Cannula for blood draws supported by evidence? An organizational case study

**DOI:** 10.1002/nop2.559

**Published:** 2020-08-05

**Authors:** Alycia Jacob, Linda Coventry, Hugh Davies, Elisabeth Jacob

**Affiliations:** ^1^ School of Nursing and Midwifery The University of Newcastle Port Macquarie NSW Australia; ^2^ School of Nursing and Midwifery Edith Cowan University Joondalup WA Australia; ^3^ School of Nursing, Midwifery and Paramedicine Australian Catholic University Brisbane QLD Australia

**Keywords:** blood sampling, cannula, evidence‐based practice, health policies, policy development, venipuncture

## Abstract

**Aim:**

To examine the quality of evidence used to inform health policies. Policies on peripheral intravenous cannulas were used as exemplars.

**Design:**

An organizational case study design was used, using the STROBE reporting guidelines.

**Methods:**

Policy guidelines were sourced between June and September 2018 from health departments in Australia. Seven documents were compared regarding intravenous cannula dwell times and blood collection use. Evidence used in the documents was critiqued using assessment guideline from the Oxford Centre for Evidence Based Medicine.

**Results:**

Large variations exist between policies regarding blood sampling and dwell time. Evidence used a variety of sources. Few references received an A evidence rating and policies differed in their interpretation of evidence.

## 1 INTRODUCTION

Sampling a person's blood is a common and frequently used clinical diagnostic procedure (Thakker et al., 2015). Blood sampling through direct venepuncture is an invasive procedure that is associated with patient discomfort and decreased satisfaction (Buowari, 2013). It is possible to reduce patent discomfort by drawing blood through an existing peripheral intravenous cannula (PIVC) and replacing PIVCs only when clinically indicated (Rickard et al., 2012). However, there is limited consensus across Australian jurisdictions as to whether (and why) the practice of drawing blood through PIVC should be supported. This paper will look at sources of evidence used to inform the creation of government health policy using the specific example of Australian state government policies on the use of PIVC for blood drawing.

## 2 BACKGROUND

In academia, the word evidence is used to refer to information that supports or disproves an hypothesis and is produced and validated through the use of specific protocols and measures (Cairney, 2016). Evidence can be taken from a variety of sources and be presented in a variety of forms, each of which will have an impact on the evidence's credibility (International Council of Nursing, 2012). Academic evidence is generally ranked according to validity of results on a scale from systematic reviews of randomized control trials as the best evidence, through to personal opinion and commentary as the least reliable evidence (Cairney, 2016). It is possible to have significant divergent opinions when deciding whether evidence exists, is relevant and should be applied to specific situations or included in public policy (Newman, Cherney, & Head, 2017).

Clinical healthcare workers often seek to incorporate evidence into health care through the use of evidence‐based practice (EBP). The term EBP is associated with the work of Archie Cochrane and his belief that clinicians should use only the most effective and proven healthcare procedures available (Mackey & Bassendowski, 2017). In clinical situations, EBP incorporates the best available evidence with the needs of health service users and the clinical skills of healthcare staff (ICN, 2012).

There can be significant delays times between the discovery of quality evidence and implementation of that evidence into clinical practice (Melnyk, Lynn, English, & Ellen, 2014). The biggest difficulty with EBP may not be creating the evidence but translating the evidence and ensuring that clinical practice is changed in favour of the evidence (ICN, 2012). Healthcare organizations may lack EBP when they are unaware of evidence‐based research or when research is not translated into policy (Stavor, Zedreck‐Gonzalez, & Hoffmann, 2017). Implementing EBP is a collective and corporate responsibility, as individual practitioners work within broader environments of teams and organizational requirements (Williams, Perillo, & Brown, 2015). It should also be acknowledged that patient care decisions are rarely made on the basis of academic and scientific evidence alone but are required to incorporate individual patient and staff factors, judgements and values (ICN, 2012).

Having healthcare policies and guidelines that are informed by current best evidence are of importance to ensure high‐quality and safe patient care. Yet there are several notable practices that continue in Australian health care in the face of high‐quality evidence. Examples include changing peripheral intravenous cannulas routinely rather than when clinically indicated and routine oxygenation of patients (Melnyk, 2017; Rickard et al., 2012).

In Australia, state governments are responsible for the operation healthcare service delivery and the creation of health policy within their jurisdictions (Dixit & Sambasivan, 2018).

It is often unclear how the concept of EBP is understood at a government policy level, as the use of evidence in policies is not always clearly referenced or identified. It is commonly alleged that policymakers are either ignorant of evidence related to their policy field or choose to ignore and fail to act on known evidence (Cairney, 2016). However, there is a specific and specialized skill set associated with evaluating large volumes of evidence and then using the results to produce recommendations (ICN, 2012). Newman et al. (2017) have argued that structural limitations within the Australian public service may have left governments unprepared to engage with research‐based and academic evidence.

Peripheral intravenous cannula (PIVC) is medical devices that are inserted into and remain in a person's peripheral vein to allow continued venous access (Wong, Cooper, Brown, Boyd, & Levinson, 2018). It is estimated that up to 80% of hospitalized patients will require intravenous therapy at some point during their inpatient hospital stay (Yagnik, Graves, & Thong, 2017). Peripheral intravenous cannula is the most commonly inserted vascular access device and can be used for administration of medications, blood sampling and management of conditions (Carr et al., 2016). Health professionals understand the importance of obtaining accurate blood sampling; however, there is considerable variability in the methods used (Bentley, Thakore, Muir, Baird, & Lee, 2016). Traditionally, most blood samples have been drawn from peripheral venepuncture, although increasingly PIVCs are being used for blood sampling (Carr et al., 2016; Davies, Coventry, Jacob, Stoneman, & Jacob, 2019).

While blood can be drawn from PIVC, there are several states and territories in Australia where this is avoided, in part, due to the perceived association between PIVC blood draws and increased risk of haemolysis, sample dilution, bloodstream infection and phlebitis (Mulloy, Lee, Gregas, Hoffman, & Ashley, 2018). Yet the evidence around the use of PIVC for blood sampling is unclear. A systematic review by Jeong et al. (2019) found that sampling from PIVC or venepuncture is likely to provide equivalent levels of accuracy for the most commonly used tests. This is in line with a systematic review by Coventry and colleagues (2019) that found limited evidence to suggest that use of PIVC for blood sampling contributed to decreased accuracy, but that PIVC use may lead to increased haemolysis rates. Relying on policies that are made based on poorly informed evidence is one of the main contributors to healthcare service failures (ICN, 2012). There are variances in practice regarding obtaining blood samples from PIVC postinsertion between individual nurses, health services and states in Australia (Davies et al., 2019). These variations and the evidence used to support them are the focus of this study. Therefore, the aim of this study is to examine what evidence is used to support Australian State government guidelines on peripheral intravenous cannula use?

## 3 METHODS

This research was conducted as an organizational case study, following the Strengthening the Reporting of Observational Studies in Epidemiology reporting guidelines (See File S1). Ethics approval was not required for this study. The authors did not have any conflict of interest to declare, and there was no funding for this project.

In June 2018, policy guidelines relating to PIVC use were sought from the relevant health departments from all Australian States and Territories. In most cases, the policies were freely available online from the health‐department websites. Where policies were not readily available, the health department in question was contacted directly and the policies were provided via email.

Seven Australian states/territories were included in the comparison. The jurisdictions were Australian Capital Territory (ACT), New South Wales (NSW), Northern Territory (NT), Queensland (QLD), Tasmania (Tas), Victoria (Vic) and Western Australia (WA). During data collection, it was found that South Australia (SA) did not have an overarching policy for the use of PIVC, but that polices were set by several local health networks. Due to the inability to access several the local health network policies, it was decided to exclude SA from this study.

The policies were assessed according to firstly, how well the policy covered the procedure of PIVC blood draws and secondly, PIVC dwell time. Data were extracted from the documents including the year the policy was published, the title of the document and whether the policy included reference to the practice of sampling of blood from a PIVC. Also extracted were details of whether blood could be drawn from a PIVC on insertion, could blood be routinely drawn from a PIVC, could a PIVC be inserted for the purpose of blood collection, was there a procedure on how to draw blood from a PIVC, were there any exceptions and did the policy outline when a PIVC should be removed or replaced.

The evidence and type of evidence that was used to support these guidelines were also extracted. To determine the type of evidence being used to justify positions taken in the guidelines, the reference lists and bibliographies of the guidelines were analysed. Data were collected relating to evidence type, where the evidence was taken from, who created the evidence and how recently it was published.

Using assessment guidelines set out by the Oxford Centre for Evidence Based Medicine (OCEBM) (2009), the choices of evidence used by the guidelines were assessed for quality. The OCEBM guidelines provide a way to categorize evidence into levels, allowing evidence in each level to be assigned a numerical value and a grade to be provided for a reference list based on the proportion or number of higher scoring items. Items are scored from 1 (Systematic Reviews of Randomized Control Trials) through to 5 (expert opinion), the number of sources from each category is identified, and a grade from A–D is applied. Allocation of an A grade indicates a strong level of evidence, while D grade indicates a weak level of evidence. The policies were also compared, to highlight possible cross‐jurisdictional inconsistencies. The grades of evidence were compared with the content of the policies to assess whether there were consistent recommendations found in guidelines that contained high levels of evidence. Two researchers independently analysed the data extracted from the policy documents. Where disagreements occurred, these were brought to the research team and discussed until consensus was reached.

## 4 FINDINGS

Data extracted from the policy documents identified large variations between policy documents in the information provided. Table 1 lists the information covered by the policies. Policies were all published between 2012–2017. Six of the documents directly referred to blood sampling from PIVCs, with three documents allowing routine collection of blood through a PIVC and three documents prohibiting collection of blood through a PIVC. Procedures for drawing blood through a PIVC differed, from specifying flushing techniques, stating only large veins to be used and whether a vacutainer could be used for withdrawal. One policy provided conflicting information, suggesting PIVCs could be used for routine blood collection and stating that blood may only be collected from PIVCs if it was specifically inserted for that reason (Tas, 2016). Some policies provided strict rules in relation to the use of PIVC for blood draws and set out specific exceptional circumstances as the only times at which they could be used (WA, 2017; QLD, 2015). The guidelines were similar in their approach to PIVC dwell time. Most Australian jurisdictions require PIVC to be replaced at 72 hr, with only Queensland allowing replacement to occur as clinically indicated.

**Table 1:** Information covered by the policies

State	Year	Document title	Specifically refers to sampling blood from PIVC	Blood may be drawn from PIVC on insertion	Blood may be routinely drawn from PIVC	PIVC may be inserted for purpose of blood collection	Procedure for PIVC blood draw specified	Specified exceptions	PIVC Dwell Time
ACT	2017	Canberra Hospital and Health Services Procedure Peripheral Intravenous Cannula, Adults and Children (Not neonates)	NO	Not stated	Not stated	Not stated	PIVC must be flushed pre‐ and postblood sampling		Must be changed after 72 hr
NSW	2013	Peripheral Intravenous Cannula (PIVC) Insertion and Post Insertion: Care in Adult Patients	Yes	Yes Only if PIVC specifically inserted for blood sampling	Yes	Yes	Aspirate via capless valve and transfer to vacutainer. Flush PIVC with 10 ml of 0.9% saline post sampling.		Must be changed after 72 hr
NT	2015	Peripheral Intravascular Catheters (PIVC) Insertion and Management (Adult) NT Health Services Procedure	Yes	Yes	No	Not stated	Blood samples may be drawn from large veins only		Must be changed after 72 hr
QLD	2015	Peripheral intravenous catheter (PIVC) Guideline	Yes	Yes	No	Not stated	Blood samples may be drawn from relatively large veins, immediately following insertion only	Blood cultures may not be taken from a PIVC	Change only when clinically indicated
Tas	2016	Peripheral Intravenous Cannula (PIVC) Insertion, care and Maintenance Protocol	Yes	Yes Not at other times unless the PIVC has been specifically inserted for blood sampling	Yes	Yes	PIVC must be flushed immediately after blood sampling. Blood samples may be taken using either syringe or vacutainer		Must be changed after 72‐ 96 hr
Vic	2012	Clinical Skills in Hospitals Project Intravenous (IV) therapy (2012) Module 1. IV Cannulation	Yes	Yes	Yes	Not stated	Bloods may be taken using a vacutainer, blue connection and interlink needle		Must be changed after 72 hr
WA	2017	Insertion and Management of Peripheral Intravenous Cannulae in Western Australian Healthcare Facilities Policy	Yes	Yes	No	No	No	Blood may only be drawn from a PIVC in an emergency; when the patient has limited vascular access or is at increased risk of bleeding or receiving thrombolytic therapy	Must be changed after 72 hr

Abbreviations: ACT, Australian Capital Territory; NSW, New South Wales; NT, Northern Territory; QLD, Queensland; Tas, Tasmania; Vic, Victoria; WA, Western Australia.

The type of evidence used by the different documents also varied (Table 2. Type of evidence used). Policies were examined to assess currency and the frequencies at which they were reviewed. Documents had publication dates between 2012–2017, all were current within the date stated for review, although one state did not list a review date. All policy documents had references cited but differed as to whether the references were cited in text, at the end of the chapter only, or in a reference list at the end of the document. The evidence used for policy development was taken from a wide variety of different sources including recent and dated academic journals, legislation/government policies, manufacturer guidelines and grey literature. Some of the state guidelines included citations for the relevant guidelines in other Australian jurisdictions, creating a circle of “self‐citing.”

**Table 2:** Types of evidence used

State	Year	Document Author(s)	Date for Review	References							Referenced in text	In text references specific for PIVC blood sampling
				Total	Academic Journals (Total)	Academic Journals (last 5 years)^a^	Legislation/ government policies/guidelines^b^	Manufacturer guidelines	Grey literature^c^	Other^d^		
ACT	2017	Health Directorate, Canberra Hospital and Health Services	2020	*N* = 15	*N* = 7 (47%)	*N* = 2 (13%)	*N* = 0 (0%)	*N* = 2 (13%)	*N* = 5 (33%)	*N* = 1 (6%)	No	No
NSW	2013	Clinical Excellence Commission	2018	*N* = 15	*N* = 4 (27%)	*N* = 0 (0%)	*N* = 5 (33%)	*N* = 0 (0%)	*N* = 5 (33%)	*N* = 1 (6%)	No	No
NT	2015	NT Clinical Guidelines Committee	2019	*N* = 12	*N* = 3 (25%)	*N* = 1 (8%)	*N* = 6 (50%)	*N* = 0 (0%)	*N* = 2 (17%)	*N* = 1 (8%)	No	No
QLD	2015	Executive Director, Communicable Diseases Branch	June 2018	*N* = 119^e^	*N* = 58 (49%)	*N* = 17 (14%)	*N* = 7 (6%)	*N* = 0 (0%)	*N* = 34 (29%)	*N* = 4 (3%)	Yes	Yes *N* = 3
Tas	2016	Quality Management Committee	2019	*N* = 23	*N* = 9 (39%)	*N* = 3 (13%)	*N* = 1 (4%)	*N* = 1 (4%)	*N* = 7 (30%)	*N* = 3 (13%)	Yes	Yes *N* = 2
Vic	2012	Department of Human Services	not stated	*N* = 3^f^	*N* = 3 (100%)	*N* = 0 (0%)	*N* = 0 (0%)	*N* = 0 (0%)	*N* = 0 (0%)	*N* = 0 (0%)	No	No
WA	2017	Communicable Disease Control Directorate	2021	*N* = 32	*N* = 14 (44%)	*N* = 6	*N* = 13	*N* = 0 (0%)	*N* = 4	*N* = 1	Yes	Yes *N* = 4

Abbreviations: ACT, Australian Capital Territory; and WA, Western Australia; NSW, New South Wales; NT, Northern Territory; QLD, Queensland; Tas, Tasmania; Vic, Victoria.

^a^ This study was conducted in 2018, so last 5yrs dates back to 2013.

^b^ Legislation was not cited exclusively by the states concerned with some policies including references to legislation from other state jurisdictions.

^c^ This Grey Literature included material published by Non‐governmental organizations and government documents below the level of legislation such as media statements

^d^ “Other” included books, websites (including some with links that were no longer valid at the time of extraction) and other sources that could not be classified in any of the specified categories.

^e^ note: the bibliography for this source stated 55 sources, but there was also an additional “reference list” with a further 66 sources. 2 were repeated, so there were a total of 119 references found.

^f^ This document contained references after each chapter, the references stated here are those that were specifically placed after the chapter entitled *Module 1: IV Cannulation*.

The level of evidence provided in each document was mapped against the OCEBM Levels of Evidence (Table 3). Identified variations in evidence are graded from categories A‐D. New South Wales and QLD both score A for their OCEBM evidence use, however, comparing the content of their policies from table 1, show significant discrepancies. These discrepancies are most apparent in the application of evidence to cannula dwell time, with QLD allowing changes to occur when clinically indicated, as opposed to NSW where they are required to be changed every 72 hr (Table 1). It is also noteworthy that the guidelines in Vic do not seem to be based on any form of evidence and received an evidence grade of D.

**Table 3:** OCEBM*Level and type of evidence

State	Academic sources (*N*)	1			2			3		4			5	Grade of recommendation
Type of Evidence		Systematic review of RCT	RCT (‐ wide confidence interval)	All or none study	Systematic review of cohort studies	Individual cohort studies; low‐quality RCT	Outcomes research; ecological studies'	Systematic review of case–control studies	Individual case–control studies	Case series	Poor quality cohort studies	Poor quality case–control studies	Expert opinion without critical appraisal	
ACT	7	2	–	–	–	2	1	–	–	–	–	–	2	Grade B
NSW	4	–	4	–	–	–	–	–	–	–	–	–	–	Grade A
NT	3	2	–	–	–	–	–	–	–	–	–	–	1	Grade B
QLD	58	9	6	–	8	9	–	1	2	4		1	22	Grade A
Tas	9	3	–	–	–	2	–	–	–	–	–	–	4	Grade B
Vic	3	–	–	–	–	1	–	–	–	–	–	–	2	Grade D
WA	14	1	3	–	–	2	1	–	–	–	1	–	6	Grade B

Note

Grade of Recommendation.

A: consistent level 1 studies.

B: consistent level 2 or 3 studies or extrapolations from level 1 studies.

C: level 4 studies or extrapolations from level 2 or 3 studies.

D: level 5 evidence or troubling inconsistent or inconclusive studies of any level.

## 5 DISCUSSION

Understanding the way that state governments view the evidence that they use when creating clinical guidelines can provide significant insight for those working in evidence creation. This study has found that many Australian state governments have endorsed policies relating to the use of peripheral intravenous cannula, including blood sampling and dwell time that are lacking, or informed to a large degree by, non‐academic evidence. The use of peripheral intravenous cannula and evidence used to support guidelines positions is inconsistent and lacks clarity.

It is intuitively known that when health policies are informed by robust and tested evidence, those policies are likely to result in more equitable and sustainable gains to population health (van de Goor et al., 2017). Organizations devoted to the promotion of evidence‐based health care have emerged in the United States and internationally, seeking to increase the production of systematic reviews and evidence synthesization (Malekinejad, Horvath, Snyder, & Brindis, 2018). The World Health Organization has long acknowledged the importance of evidence in health through an advisory committee dedicated to health research and set up a sub‐committee specifically to examine the use of research evidence (Whitworth, 2006). While evidenced‐based policy may be a positive goal to aim for, seeking complete evidence integration into government policy may be seen as naïve and potentially unobtainable (Cairney, 2016). It is more reasonable to expect that policy could be informed by evidence than based on it, as feasibility, equity and politics will routinely be factored into decision‐maker process (Whitworth, 2006).

Governmental policies do not always clearly describe the evidence that was used in their development or outline the process that was taken to incorporate evidence into the directions and guidelines (van de Goor et al., 2017). This study was a reverse of the traditional method of policy investigation, whereby the researchers looked at the policies to find the evidence supporting the use of a particular clinical practice, as opposed to looking at the evidence to ascertain whether it is followed. Because only two of the seven Australian jurisdictions received an evidence rating of A and those policies differed in their interpretation of the evidence that they did use, it is quite clear that there is more to be done in the promotion of research into the use of PIVC for blood draws.

van de Goor et al. (2017) conducted a study of the reasons why European countries struggled to integrate evidence into their health policies. They found that having a lack of concrete evidence in specific areas, having a lack of existing evidence on the cost of specific procedures or programmes and lacking a joint and shared understanding of the barriers to implementation were significant reasons for reduced policy evidence uptake. More rigorous policy creation processes may be required to allow readers to understand the inconsistencies between policies, and the way evidence is used in policy creation and development (Oxman, Fretheim, Schünemann, & SURE, Fretheim & Schünemann, 2006).

It may seem intuitive to suggest that policymakers should make policy based on evidence; however, a strict application of evidence would remove the mandate of decision‐making from those who were elected as the policymaker and decision‐maker (Cairney, 2016). Healthcare decision‐makers are often interested in using evidence to support their decision‐making but may find that the scientific evidence available is inadequate for their needs (Tunis, Stryer, & Clancy, 2003). When making health policy decisions, decision‐makers must navigate not just the scientific evidence, but also the intangible and politically sensitive concepts of value judgement and public acceptability of actions (Mays, Pope, & Popay, 2005). Policymakers need evidence from a wide range of sources and often find that this evidence comes from personal contacts and individual expert sources (van de Goor et al., 2017).

Policymakers are also often required to create policies in areas where they are not experts and in situations where ambiguity around the scientific basis for specific actions exist (Cairney, 2016). Black and Donald (2001) provide an example of the different ways that government policies may interpret evidence, showing the considerable variation in the international guidelines for cholesterol testing. These guidelines are based on identical evidence, and the variations can be attributed to differences in value judgements. Even among academic circles, there can be significant discrepancies in how complex scientific evidence is understood and interpreted (Black & Donald, 2001).

In the case of drawing blood from PIVCs, some government guidelines have set specific actions either prohibiting or encouraging blood draws. These stances are held despite a recent systematic review (Coventry et al., 2019) recommending that further research is required to inform the evidence for best practice recommendations to evaluate whether blood samples are similar if obtained by PIVC compared with venepuncture. The gaps in evidence around the use of PIVCs may be a related to the lack of emphasis placed among research bodies on conducting clinical trials in a way that can meet the needs of decision‐making bodies (Tunis et al., 2003).

A recent cross‐sectional survey of nurses from across Australia failed to find a consensus between clinical professionals on whether taking blood from PIVC was acceptable practice, including within‐state jurisdictions that have specified policies on PIVC blood taking (Davies et al., 2019). It is interesting to note that Black and Donald (2001) have suggested that increased involvement of clinical healthcare workers in policy creation may actually result in policies that contain lower levels of evidence. It may be that many senior nurses who have responsibility for policy development do not have the required academic skills to use the best available research. The implications for nurses and healthcare workers who are looking to improve the evidence base of the policies that they are working within are that may benefit from education and upskilling specifically related to understanding evidence and research capacity (Lee et al., 2020).

### 5.1 Limitations

This paper looked at the evidence that was specifically cited in policies. There may have been additional evidence that was used to support the creation of policies, particularly expert opinions that may not have been referenced in the policies and thus could not be included.

### 5.2 Recommendations

As current guidelines do not appear to use high levels of evidence, policymakers should give weight to the importance of academic rigour in the creation of clinical policies and guidelines. State governments should ensure that searches are conducted for academic evidence in support (or opposition) of specific practices prior to guideline publication. Policymakers may wish to consider providing grades of evidence to support specific policies, allowing for more open discussion over where new evidence is required.

Where there is clear evidence for a clinical policy, such as dwell time for PIVCs, decision‐makers appear to be ignoring the evidence. State governments should consider employing people with academic skills and clinical experience in related professions in advisory roles prior to publication of health policy documents to review the evidence.

Involving clinicians and clinically trained academics in policy creation could assist with reducing the divide between policy and practice, as well as providing a conduit for communicating guidelines to clinical professionals. Future research could consider the capacity of clinical staff to engage with varying levels of evidence and the upskilling required to assist clinicians to think critically about knowledge translation.

## 6 CONCLUSION

The use of high‐quality evidence in the creation of health policy is important to ensure that the policy results in positive health outcomes. Health policy guidelines for the use of PIVC in blood sampling in Australia are inconsistent and inconsistently supported by evidence. While it may not always be feasible for decision‐makers to base policies on evidence, they should be at the least informed by evidence. Barriers to evidence‐based policy can include lack of available or consistent evidence and lack the skills of skills by decision‐makers to understand academic level clinical information. Governments should be at the forefront for using research‐based evidence where available instead of informing themselves through the expert opinions and individual contacts.

## CONFLICT OF INTEREST

The authors declare that they have no conflicts of interest associated with this study.

## AUTHOR CONTRIBUTIONS

AJ and EJ: Study design. AJ: Data extraction. EJ, AJ, HD and LC: Methodology. AJ and LC: Data analysis. AJ, HD, LC and EJ all contributed towards editing and all authors approved the final manuscript.

## Supporting information

Supplementary Material
